# Liquid-liquid phase separation-associated gene networks link EMT and ferroptosis to define a PDGFRB-centered prognostic signature in bladder cancer

**DOI:** 10.3389/fimmu.2026.1846096

**Published:** 2026-06-24

**Authors:** Ximo Zhang, Peng Ge, Qiwei Chen, Shujing Wang, Deyong Yang

**Affiliations:** 1 Department of Urology, The First Affiliated Hospital of Dalian Medical University, Dalian, China; 2 Department of Interventional Therapy, The First Affiliated Hospital of Dalian Medical University, Dalian, China; 3 Department of Biochemistry and Molecular Biology, Institute of Glycobiology, Dalian Medical University, Dalian, China

**Keywords:** bladder cancer, epithelial-mesenchymal transition, ferroptosis, liquid-liquid phase separation, PDGFRB, prognostic signature

## Abstract

**Introduction:**

Bladder cancer (BLCA) remains a formidable clinical challenge due to its high recurrence rate and complex molecular landscape. The integrated roles of liquid-liquid phase separation (LLPS), epithelial-mesenchymal transition (EMT), and ferroptosis in BLCA progression remain largely unresolved.

**Methods:**

We integrated bulk RNA-seq, single-cell RNA sequencing (scRNA-seq), and spatial transcriptomics to develop a 5-gene prognostic signature. Functional experiments including Western blot, transmission electron microscopy, lipid peroxidation assays, and Ferrostatin-1 rescue were performed in T24 bladder cancer cells. Tissue validation was conducted in clinical specimens and a mouse orthotopic model.

**Results:**

We identified a 5-gene signature (PDGFRB, FLNA, JUN, SNAI2, and ITGB1) that effectively stratifies BLCA patients into distinct risk groups. PDGFRB-positive epithelial subsets were characterized by high LLPS-associated gene expression and dispersed spatial localization. Immunofluorescence revealed endogenous PDGFRB condensate-like puncta in both human and mouse bladder cancer tissues. *In vitro* reconstitution confirmed the phase separation capacity of the PDGFRB intrinsically disordered region. PDGFRB knockdown impaired cell proliferation and migration, and induced ferroptosis as demonstrated by mitochondrial damage, elevated lipid peroxidation, and rescue by Ferrostatin-1. Immune infiltration analysis showed that the five genes, particularly PDGFRB and FLNA, were significantly associated with immune and stromal scores.

**Discussion:**

These findings define a PDGFRB-centered prognostic signature that serves as a transcriptomic framework linking LLPS-associated gene networks with EMT and ferroptosis vulnerability. The study provides experimental evidence supporting PDGFRB as a potential therapeutic target and suggests implications for the tumor immune microenvironment in bladder cancer.

## 1 Introduction

Bladder cancer (BLCA) remains a formidable global health challenge characterized by high recurrence rates and poor prognosis in advanced stages (1). Despite the clinical implementation of immunotherapy and targeted agents, outcomes for patients with metastatic disease remain unsatisfactory (2, 3). While early-stage non-muscle-invasive disease can be managed with transurethral resection and intravesical therapy, recurrence rates remain high, and up to 21% of patients with high-risk disease progress to muscle-invasive bladder cancer, which is associated with poor prognosis (4). Recent advances in molecular subtyping have refined the understanding of BLCA heterogeneity, yet the translation of these classifications into targeted therapeutic strategies remains limited (5, 6).

Liquid-liquid phase separation (LLPS) has recently emerged as a fundamental biophysical principle governing the formation of membrane-less organelles and biomolecular condensates (7, 8). By concentrating proteins and nucleic acids into dynamic compartments, LLPS facilitates the spatiotemporal regulation of critical cellular processes, including transcription and signal transduction (8, 9). The physical chemistry of LLPS is driven by multivalent weak interactions that are frequently mediated by intrinsically disordered regions (IDRs), and the phase behavior of proteins is highly sensitive to environmental conditions such as molecular crowding, salt concentration, and post-translational modifications (10–13). Aberrant LLPS is increasingly implicated in cancer pathogenesis, where altered phase separation can disrupt gene expression programs, deregulate signaling networks, and promote oncogenic transformation (14, 15). IDR-containing proteins have been demonstrated to participate in phase separation-mediated transcriptional regulation and signal transduction, forming condensates that function as signaling hubs to coordinate oncogenic programs (16, 17). Notably, phase separation is not limited to soluble proteins; recent evidence demonstrates that transmembrane proteins can also undergo LLPS at membrane surfaces, as exemplified by PDZD8-mediated condensate formation on the endoplasmic reticulum (18).

Platelet-derived growth factor receptor beta (PDGFRB), a member of the receptor tyrosine kinase family, has emerged as a critical player in bladder cancer (BLCA) progression and therapy resistance. PDGFRB signaling activates several downstream pathways, including the PI3K/Akt and Ras/MAPK cascades, which collectively drive proliferation, survival, and migration (19, 20). Early studies demonstrated that PDGFRB expression drives resistance to epidermal growth factor receptor (EGFR)-targeted therapies. Mechanistically, this resistance is mediated by the formation of EGFR-PDGFRB heterodimers, which sustain downstream mitogen-activated protein kinase (MAPK) signaling even in the presence of EGFR inhibitors, thereby bypassing therapeutic blockade and maintaining tumor cell proliferation and invasion (21, 22). Beyond its role in acquired resistance, PDGFRB also functions as a key regulator of cancer stem-like cell (CSC) maintenance. A YAP/TEAD1/PDGF-BB/PDGFRB autocrine positive feedback loop has been identified, wherein PDGFRB activation stabilizes YAP and promotes its nuclear translocation, which in turn drives PDGFB transcription and sustains CSC self-renewal, tumor initiation, and cisplatin resistance (23). Given that both therapeutic resistance and stemness are tightly linked to epithelial-mesenchymal transition (EMT) and metabolic reprogramming, PDGFRB may serve as a central molecular hub integrating these malignant phenotypes.

Among these programs, the epithelial-mesenchymal transition (EMT) serves as a major driver of metastasis and therapeutic resistance (24, 25). EMT is not a binary switch but rather a dynamic spectrum of intermediate states, which confer enhanced plasticity and adaptive capacity to carcinoma cells (26, 27). EMT is orchestrated by a network of transcription factors, such as SNAI2 and JUN, which repress epithelial markers to activate mesenchymal phenotypes (28). Recent evidence suggests that LLPS provides a localized regulatory platform for these factors, thereby modulating the plastic transition of cancer cells (29). Concurrently, ferroptosis, an iron-dependent form of regulated cell death driven by lipid peroxidation, has emerged as a promising strategy to eliminate therapy-resistant cells (30, 31). Characterized by mitochondrial shrinkage and glutathione depletion, ferroptosis is sensitive to both metabolic and mechanical cues (32, 33). However, whether PDGFRB signaling interfaces with LLPS to modulate ferroptotic vulnerability in bladder cancer remains to be elucidated.

Intriguingly, recent studies have revealed functional intersections between LLPS, EMT, and ferroptosis. EMT states can confer ferroptosis resistance in specific contexts (34). LLPS has been shown to directly regulate ferroptotic vulnerability by modulating the activity of key proteins like FSP1 (35). As discussed above, PDGFRB has been independently implicated in driving EMT-associated drug resistance and maintaining cancer stemness in bladder cancer, yet its potential role at the intersection of these three processes has not been explored. In this study, we integrated bulk, single-cell, and spatial transcriptomics to identify a 5-gene prognostic signature (PDGFRB, FLNA, JUN, SNAI2, ITGB1). Through multi-omics analysis, functional validation, and *in vitro* phase separation assays, we establish PDGFRB as a central node linking LLPS, EMT, and ferroptosis, and identify it as a critical therapeutic target in bladder cancer.

## 2 Materials and methods

### 2.1 Data collection and processing

Transcriptomic data and clinical information for bladder cancer (BLCA) patients were obtained from The Cancer Genome Atlas (TCGA) database (36). Single-cell RNA sequencing (scRNA-seq) data (GSE222315) and spatial transcriptomics data (GSE247629) were downloaded from the Gene Expression Omnibus (GEO) database (37). For external validation of the prognostic signature, an independent BLCA cohort (GSE13507) with clinical survival metadata was obtained from the GEO database (38). Ferroptosis-related genes were retrieved from FerrDb V3 (39), EMT-related genes from MSigDB (40), and LLPS-related genes from DrLLPS (41). All analyses were performed using R software (version 4.2.0).

### 2.2 Identification of core genes and prognostic signature

Differentially expressed genes between tumor and normal tissues were identified using the “limma” R package (42). Venn diagram analysis was performed to identify intersecting genes among LLPS, EMT, and ferroptosis gene sets. Univariate Cox regression analysis was conducted to evaluate prognostic significance. LASSO Cox regression with ten-fold cross-validation was used to construct the prognostic signature (43). Patients were divided into high-risk and low-risk groups based on the median risk score. Kaplan-Meier survival analysis was performed using the log-rank test, and time-dependent ROC curves were constructed using the “timeROC” R package (44). For external validation, risk scores were calculated for each patient in the GSE13507 cohort using the gene coefficients derived from the TCGA training set, and Kaplan-Meier and ROC analyses were performed using the same methods as described above.

### 2.3 Single-cell and spatial transcriptomics analysis

scRNA-seq data were analyzed using the “Seurat” R package (45). Cells with >20% mitochondrial content were excluded. Data were normalized, and batch effects were corrected using Harmony (46). Cell clusters were identified using PCA and UMAP, and cell types were annotated based on canonical markers. Pseudotime trajectory analysis was performed using Monocle2 (47). Spatial transcriptomics data were analyzed using Seurat, and spatial feature plots were generated using SpatialFeaturePlot. The LLPS-associated gene expression score (LLPS-assoc. gene score) was calculated as the mean expression of the five core prognostic genes (PDGFRB, FLNA, JUN, SNAI2, ITGB1). The EMT score and ferroptosis score were similarly computed as the mean expression of the EMT-related gene set (from MSigDB) and the ferroptosis-related gene set (from FerrDb V3), respectively. For bulk tissue analysis, the enrichment score of LLPS-associated genes was calculated using ssGSEA implemented in the GSVA package with the full DrLLPS gene set.

### 2.4 Immune cell infiltration

For each BLCA sample, the ESTIMATE method (48) was used to calculate the immunological score, the stromal score, and the estimated score. The CIBERSORT method (49) was used in conjunction with the LM22 signature matrix to calculate the relative proportions of 22 tumor-infiltrating immune cell types. Furthermore, the ssGSEA method implemented in the GSVA package (50) was used to assess the enrichment levels of 24 immune cell groups using immune-related gene sets retrieved from the MSigDB database. Correlation analyses were conducted to investigate the associations between critical prognostic genes, immune cells, and immune-related scores.

### 2.5 Cell culture

The human bladder cancer cell line T24 was obtained from the Cell Bank of Type Culture Collection of the Chinese Academy of Sciences. Cell line identity was authenticated by short tandem repeat (STR) profiling. Cells were cultured in RPMI-1640 medium (Gibco, Grand Island, NY, USA) supplemented with 10% fetal bovine serum and 1% penicillin-streptomycin at 37 °C with 5% CO_2_. Ferrostatin-1 (Fer-1, Targetmol, #347174-05-4) was purchased from Targetmol and dissolved in DMSO.

### 2.6 Clinical specimens

Formalin-fixed, paraffin-embedded (FFPE) tumor tissues from six patients with primary bladder cancer (three low-grade and three high-grade) were obtained from the archives of the First Affiliated Hospital of Dalian Medical University. All specimens were originally collected from a previously established institutional cohort (April 2022 to January 2024, n = 42; ethical approval No. PJ-KS-KY-2023-352). Histological grading was independently confirmed by two urological pathologists, and all patients had provided written informed consent for the use of residual surgical material in research.

### 2.7 Animal studies

All animal procedures were approved by the IACUC of Dalian Medical University (approval No. AEE24324). Orthotopic bladder tumors were established in BALB/c nude mice by injecting T24 cells into the bladder wall. For the present study, archived paraffin sections containing both tumor and matched adjacent normal urothelium from untreated control animals (n = 3) were retrieved from a prior xenograft experiment and used for immunofluorescence analysis.

### 2.8 Tissue immunofluorescence staining

Paraffin-embedded tissue sections (4 μm thickness) were deparaffinized in xylene and rehydrated through a graded ethanol series. Antigen retrieval was performed in citrate buffer (pH 6.0) using a microwave. After cooling, sections were blocked with 5% bovine serum albumin (BSA) for 1 hour at room temperature and then incubated with primary antibody against PDGFRB (Proteintech, #13449-1-AP, 1:200) overnight at 4 °C. Following three washes with PBS, slides were incubated with Alexa Fluor-conjugated secondary antibody for 1 hour at room temperature in the dark. Nuclei were counterstained with DAPI. After mounting, images were captured using a fluorescence microscope and scanned for further analysis.

### 2.9 Recombinant protein expression and purification

The DNA sequence encoding the C-terminal intrinsically disordered region of human PDGFRB (residues 951–1089, predicted by PONDR-VSL2 (51)) was synthesized with codon optimization for E. coli and cloned into the pET-28a-EGFP vector to generate an N-terminal His_6_-EGFP fusion protein (EGFP-PDGFRB-IDR). The construct was verified by sequencing. The expression plasmid was transformed into E. coli BL21(DE3). Cells were grown in LB medium containing 50 μg/mL kanamycin at 37 °C to an OD_600_ of 0.6–0.8, and protein expression was induced with 0.2 mM IPTG at 16 °C for 16–20 h. Bacteria were harvested, lysed by sonication, and the recombinant protein was purified using Ni−NTA affinity chromatography followed by size−exclusion chromatography on a Superdex 200 Increase column pre−equilibrated with storage buffer (20 mM HEPES, pH 7.5, 150 mM NaCl, 1 mM DTT). The purified protein was concentrated, snap−frozen, and stored at −80 °C.

### 2.10 *In vitro* droplet formation assay

Purified EGFP−PDGFRB−IDR was diluted to final concentrations of 1, 2.5, 5, 10, and 20 μM in a phase−separation buffer containing 20 mM HEPES (pH 7.5), 150 mM NaCl, 1 mM DTT, and 5% (w/v) polyethylene glycol 8000 (PEG−8000). After incubation at room temperature for 10 min, each sample was transferred to a glass−bottom confocal dish and imaged on a Leica TCS SP8 confocal microscope using a 63× oil−immersion objective with 488−nm excitation.

### 2.11 Lentivirus-mediated shRNA knockdown

The pLKO.1 shRNA lentivirus system was used to generate shRNA lentivirus targeting human PDGFRB. The target sequence for PDGFRB shRNA is 5′-CACCATTCCATGCCGAGTAAC-3′. A non-targeting scramble shRNA was used as the negative control (shNC). T24 cells at 30% confluence on a 6-cm dish were transfected with PDGFRB shRNA lentivirus using 6 μg/mL polybrene (TR-1003, Sigma-Aldrich) to enhance transduction efficiency. PDGFRB-knockdown (shPDGFRB) cells were obtained by puromycin screening (2 μg/mL) in T24 cells. Knockdown efficiency was confirmed by immunofluorescence staining prior to functional assays.

### 2.12 Western blot analysis

Cells were lysed in RIPA buffer (Beyotime, #P0013B) supplemented with protease and phosphatase inhibitors. Protein concentrations were determined using a BCA assay kit (Beyotime, #P0010). Equal amounts of protein (30 μg) were separated by 8% SDS−PAGE and transferred onto PVDF membranes (Millipore, #IPVH00010). Membranes were blocked with 5% non−fat milk in TBST for 1 h at room temperature and incubated overnight at 4 °C with primary antibody against PDGFRB (Bioworld, #BS45283, 1:1000) and β−actin (Proteintech, #66009−1−Ig, 1:5000). After washing, membranes were incubated with HRP−conjugated secondary antibodies for 2 h at room temperature. Protein bands were visualized using enhanced chemiluminescence (ECL) reagent (Beyotime, #P0018) and imaged with a ChemiDoc imaging system (Bio−Rad).

### 2.13 Cell viability assay

Cell viability was assessed using the Cell Counting Kit-8 (CCK-8; ApexBio, #K101831133EF5E). Cells were seeded in 96-well plates at a density of 3 × 10³ cells per well. After treatment, 10 μL of CCK-8 solution was added to each well and incubated for 2 hours at 37 °C. Absorbance was measured at 450 nm using a microplate reader.

### 2.14 Wound healing assay

Cells were seeded in 6-well plates and cultured to 90-100% confluence. A sterile 200 μL pipette tip was used to create a scratch wound across the monolayer. Cells were washed twice with PBS and cultured in serum-free medium. Images were captured at 0 and 24 hours using an inverted microscope. Wound closure rate was quantified by measuring the remaining wound area.

### 2.15 Transmission electron microscopy

Cells were fixed with 2.5% glutaraldehyde, post-fixed with 1% osmium tetroxide, dehydrated, and embedded in Epon resin. Ultrathin sections (70 nm) were stained with uranyl acetate and lead citrate and examined under a transmission electron microscope.

### 2.16 Lipid peroxidation assay

Lipid peroxidation was measured using C11-BODIPY 581/591 (Thermo Fisher Scientific, #D3861). Cells were incubated with 2 μM C11-BODIPY in PBS for 30 minutes at 37 °C, and fluorescence was analyzed by flow cytometry. Mean fluorescence intensity was calculated to quantify lipid peroxidation levels.

### 2.17 Malondialdehyde assay

Malondialdehyde (MDA) levels were measured using a commercial Lipid Peroxidation MDA Assay Kit (Beyotime, #S0131S) following the manufacturer’s protocol. Cells were lysed and incubated with thiobarbituric acid solution at 100 °C for 15 minutes. Absorbance was measured at 532 nm and normalized to protein concentration.

### 2.18 Statistical analysis

All statistical analyses for *in vitro* experiments were performed using GraphPad Prism software (version 10.0). Data are presented as mean ± standard deviation (SD). Differences between two groups were assessed using unpaired two-tailed Student’s t-test. Comparisons among three or more groups were analyzed by one-way analysis of variance (ANOVA). For experiments involving two independent variables (e.g., time and treatment), two-way ANOVA was used. A p-value of less than 0.05 was considered statistically significant.

## 3 Results

### 3.1 Identification of LLPS-EMT-ferroptosis core genes in BLCA

**Figure 1:**
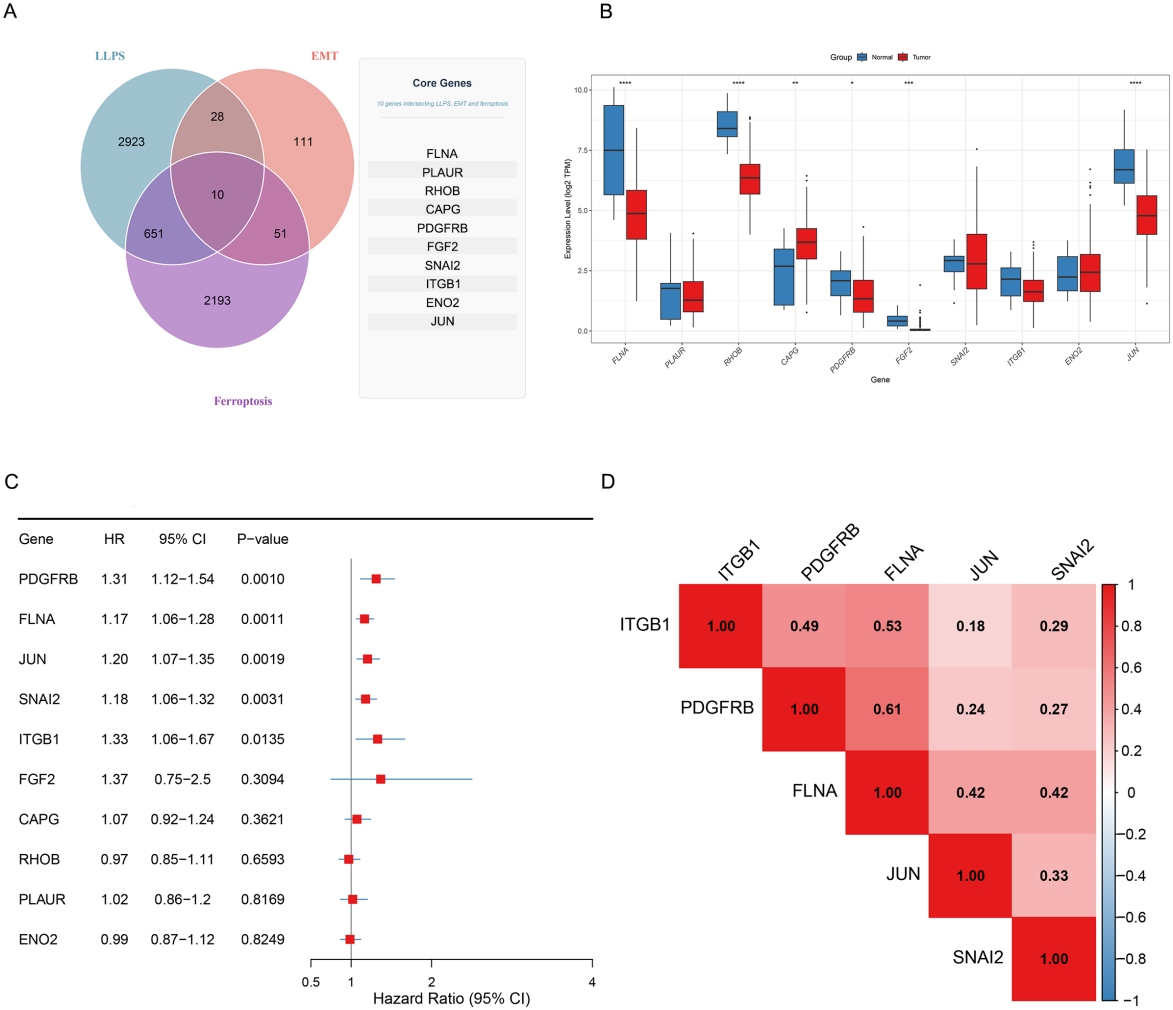
Identification of LLPS-EMT-ferroptosis core genes in BLCA. **(A)** Venn diagram showing the intersection of EMT-related genes (n = 200), ferroptosis-related genes (n = 2, 905), and LLPS-related genes (n = 3, 612). Ten genes were identified as core LLPS-EMT-ferroptosis genes. **(B)** Box plots showing expression levels of the 10 core genes in BLCA tumor tissues (n = 412) compared to normal tissues (n = 19). Statistical significance was determined by Student’s t-test. *p < 0.05, **p < 0.01, ***p < 0.001, ****p < 0.0001. **(C)** Forest plot of univariate Cox regression analysis for the 10 core genes. Hazard ratios (HR) and 95% confidence intervals are shown. Genes with p < 0.05 (PDGFRB, FLNA, JUN, SNAI2, ITGB1) are indicated. **(D)** Correlation heatmap of the five prognostic genes. The color scale represents Pearson correlation coefficients, with red indicating positive correlation and blue indicating negative correlation. Numbers in the upper triangle indicate the correlation coefficients.

To identify key molecular drivers in bladder cancer (BLCA), we intersected gene sets associated with liquid-liquid phase separation (LLPS), epithelial-mesenchymal transition (EMT), and ferroptosis. A Venn diagram identified 10 core genes common to all three biological processes (**Figure 1A**). Differential expression analysis between TCGA-BLCA tumor tissues (n = 412) and normal tissues (n = 19) revealed that most of these core genes were significantly dysregulated in malignancy (**Figure 1B**). Subsequently, univariate Cox regression analysis was performed to evaluate the prognostic significance of these candidates. Five genes—PDGFRB, FLNA, JUN, SNAI2, and ITGB1—exhibited hazard ratios (HR) greater than 1 with p-values < 0.05, identifying them as key risk factors for poor survival (**Figure 1C**). Pearson correlation analysis further demonstrated a strong positive inter-correlation among these five prognostic genes (**Figure 1D**), suggesting they may function as a coordinated regulatory module in the progression of BLCA.

### 3.2 Construction of the 5-gene LLPS-EMT-ferroptosis prognostic signature

**Figure 2:**
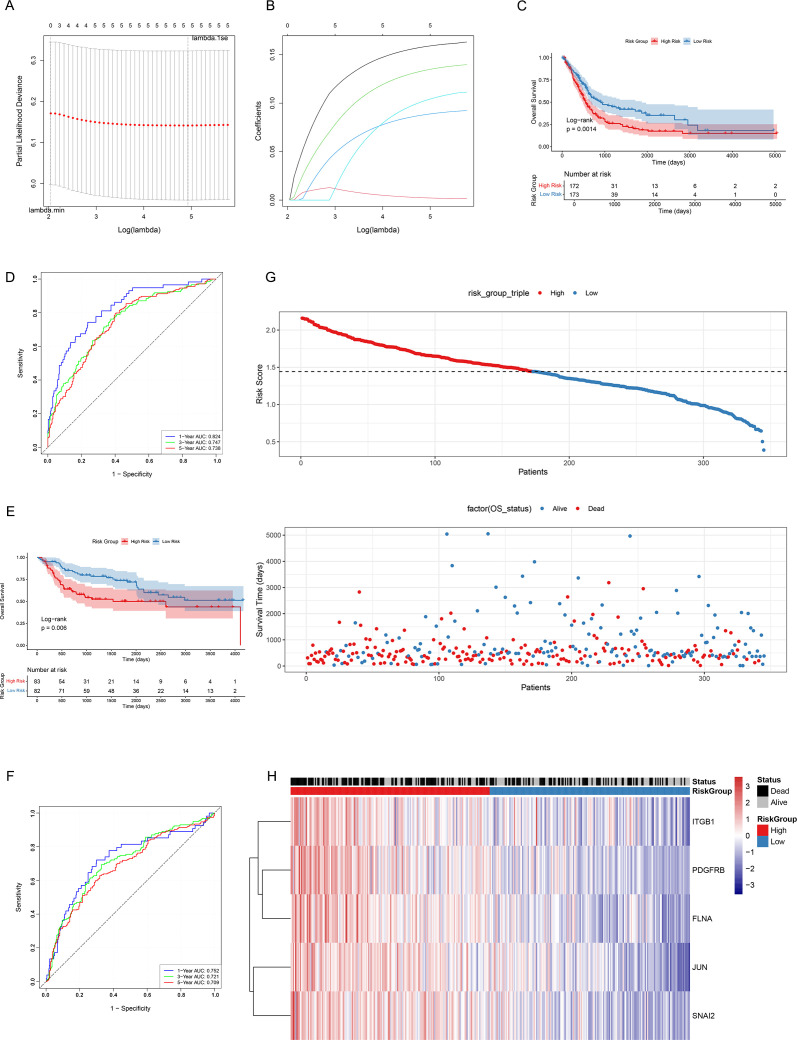
Construction and validation of the 5-gene LLPS-EMT-ferroptosis prognostic signature in BLCA. **(A)** Cross-validation plot for tuning parameter selection in LASSO regression analysis. The optimal λ values (λ.min, left vertical dashed line; λ.1se, right vertical dashed line) are indicated. **(B)** LASSO coefficient paths for the five genes. All five genes were retained with non-zero coefficients at λ.min. **(C)** Kaplan-Meier survival curves for high-risk (n = 172) and low-risk (n = 173) groups based on the 5-gene signature in the TCGA-BLCA training cohort. The log-rank p-value is shown. Shaded areas represent 95% confidence intervals. **(D)** Time-dependent ROC curves for 1-, 3-, and 5-year survival prediction in the TCGA-BLCA training cohort. AUC values are shown in the legend. **(E)** Kaplan-Meier survival curves for high-risk and low-risk groups in the external validation cohort (GSE13507, n = 165). The log-rank p-value is shown. **(F)** Time-dependent ROC curves for 1-, 3-, and 5-year survival prediction in the external validation cohort (GSE13507). AUC values are shown in the legend. **(G)** Distribution of risk scores (top) and survival status (bottom) of 345 BLCA patients in the TCGA-BLCA cohort. Patients are ranked by increasing risk score, with the median value used as the cutoff to divide them into high-risk (red) and low-risk (blue) groups. **(H)** Heatmap showing expression patterns of the five model genes across the 345 patients in the TCGA-BLCA cohort. Rows represent genes, columns represent patients. Red indicates high expression, blue indicates low expression. Top annotation bars indicate risk group (red = high, blue = low) and survival status (black = dead, gray = alive). The dendrogram on the left indicates clustering of genes with similar expression patterns, with PDGFRB and FLNA showing the closest relationship.

Based on the five prognostic genes identified from univariate Cox analysis (PDGFRB, FLNA, JUN, SNAI2, ITGB1), we constructed a prognostic signature using LASSO Cox regression analysis. The optimal λ values were determined by ten-fold cross-validation (**Figure 2A**), and all five genes were retained with non-zero coefficients (**Figure 2B**). Using the median risk score as the cutoff, 345 BLCA patients were divided into high-risk (n = 172) and low-risk (n = 173) groups. Kaplan-Meier analysis demonstrated significantly worse overall survival in the high-risk group (**Figure 2C**). Time-dependent ROC curves demonstrated favorable predictive performance of the signature (**Figure 2D**). To assess the generalizability of our prognostic signature, we performed external validation in an independent BLCA cohort (n = 165). Using the same gene coefficients derived from the TCGA training set, risk scores were calculated for each patient in the external cohort, and patients were stratified by the median risk score. Kaplan-Meier analysis confirmed that the high-risk group exhibited significantly worse overall survival (**Figure 2E**). Time-dependent ROC analysis further corroborated the prognostic value of the signature in the external cohort (**Figure 2F**). These results demonstrate that the prognostic value of our signature extends beyond the training dataset and is reproducible in an independent patient population. Risk score distribution analysis showed that patients with higher risk scores had shorter survival times and higher mortality rates (**Figure 2G**). Heatmap visualization revealed coordinated upregulation of all five genes in high-risk patients (**Figure 2H**). Collectively, these results demonstrate that the 5-gene signature effectively stratifies BLCA patients by prognosis in both internal and external cohorts, providing a robust tool for predicting patient outcomes.

### 3.3 Assessment of LLPS potential and functional profiling of the core genes

**Figure 3:**
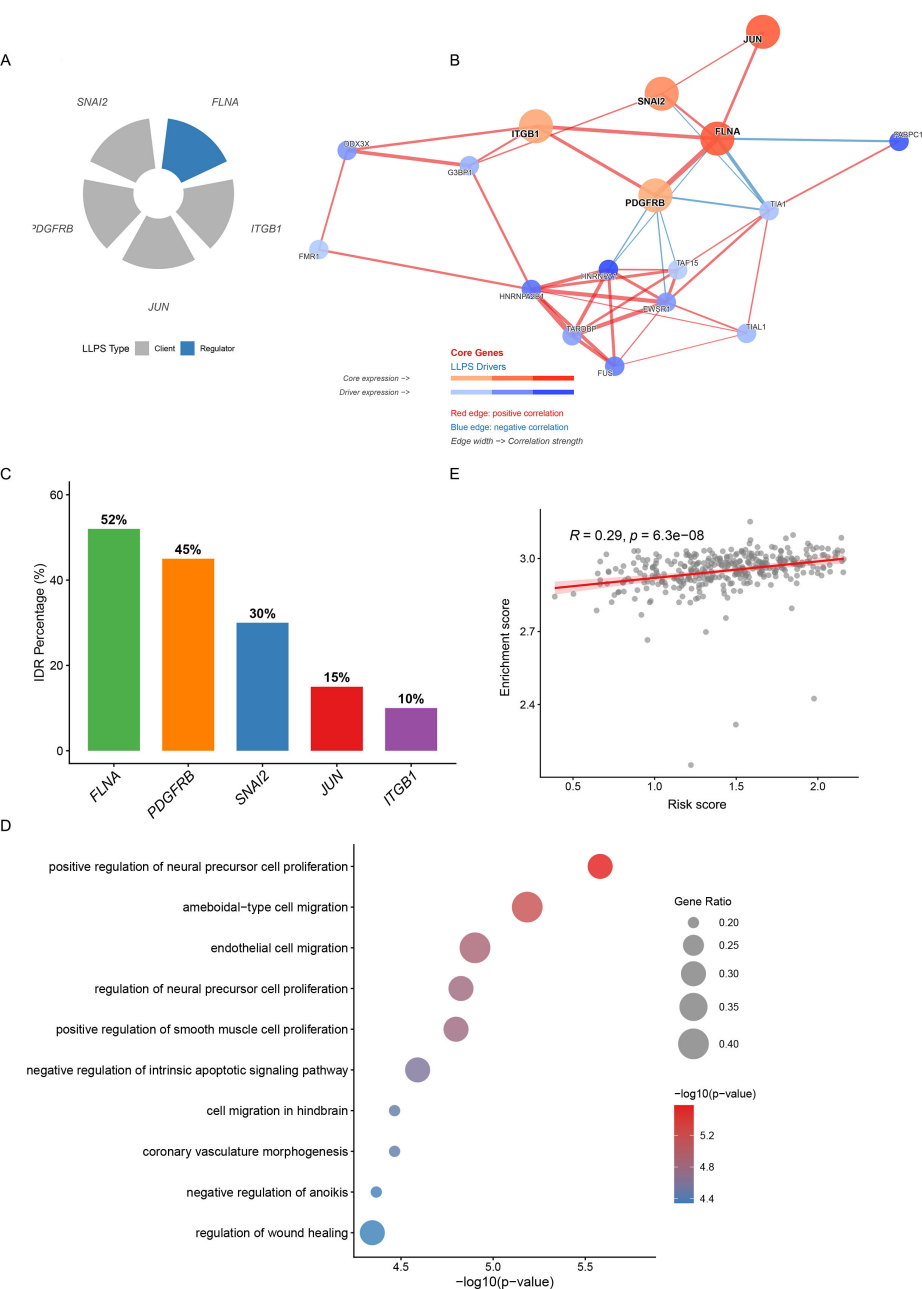
Assessment of LLPS potential of the 5 core genes. **(A)** Classification of the five core genes in the DrLLPS database. FLNA is annotated as a regulator, while JUN, SNAI2, ITGB1, and PDGFRB are classified as clients, indicating their involvement in LLPS through interactions with scaffold proteins. **(B)** Co-expression network of the five core genes with known LLPS driver proteins. Red nodes represent core genes, blue nodes represent LLPS drivers. Edge thickness indicates correlation strength; red edges indicate positive correlation, blue edges indicate negative correlation. **(C)** Intrinsically disordered region (IDR) content of the five core genes. FLNA and PDGFRB exhibit high IDR percentages (52% and 45%, respectively), while SNAI2, JUN, and ITGB1 show lower IDR content (30%, 15%, and 10%, respectively). **(D)** GO enrichment analysis of the five core genes, highlighting terms related to cell migration, regulation of cell proliferation, and negative regulation of apoptotic signaling. **(E)** Correlation between the enrichment score of LLPS-associated genes (calculated by ssGSEA using all DrLLPS genes) and the risk score. A significant positive correlation was observed (r = 0.29, p = 6.3 × 10^-8^).

To investigate the LLPS-associated features of the five core genes, we evaluated their involvement in LLPS networks using the DrLLPS database. FLNA is annotated as a regulator, while JUN, SNAI2, ITGB1, and PDGFRB are classified as clients, suggesting they participate in LLPS through interactions with scaffold proteins (**Figure 3A**). Co-expression analysis further revealed extensive interactions between these five core genes and known LLPS driver proteins, supporting their integration into complex signaling condensates (**Figure 3B**). Notably, protein sequence analysis demonstrated that FLNA and PDGFRB possess high percentages of intrinsically disordered regions (IDRs) at 52% and 45%, respectively, providing a structural basis for their phase-separating capacity (**Figure 3C**). GO enrichment analysis indicated that these genes are primarily involved in cell migration, regulation of cell proliferation, and negative regulation of apoptotic signaling, which are processes associated with tumor progression and metastatic potential (**Figure 3D**). Finally, we observed a significant positive correlation between the enrichment score of LLPS-associated genes and the risk score (**Figure 3E**), confirming that elevated LLPS potential is a hallmark of high-risk BLCA patients.

### 3.4 Single-cell landscape and correlation between LLPS and EMT

**Figure 4:**
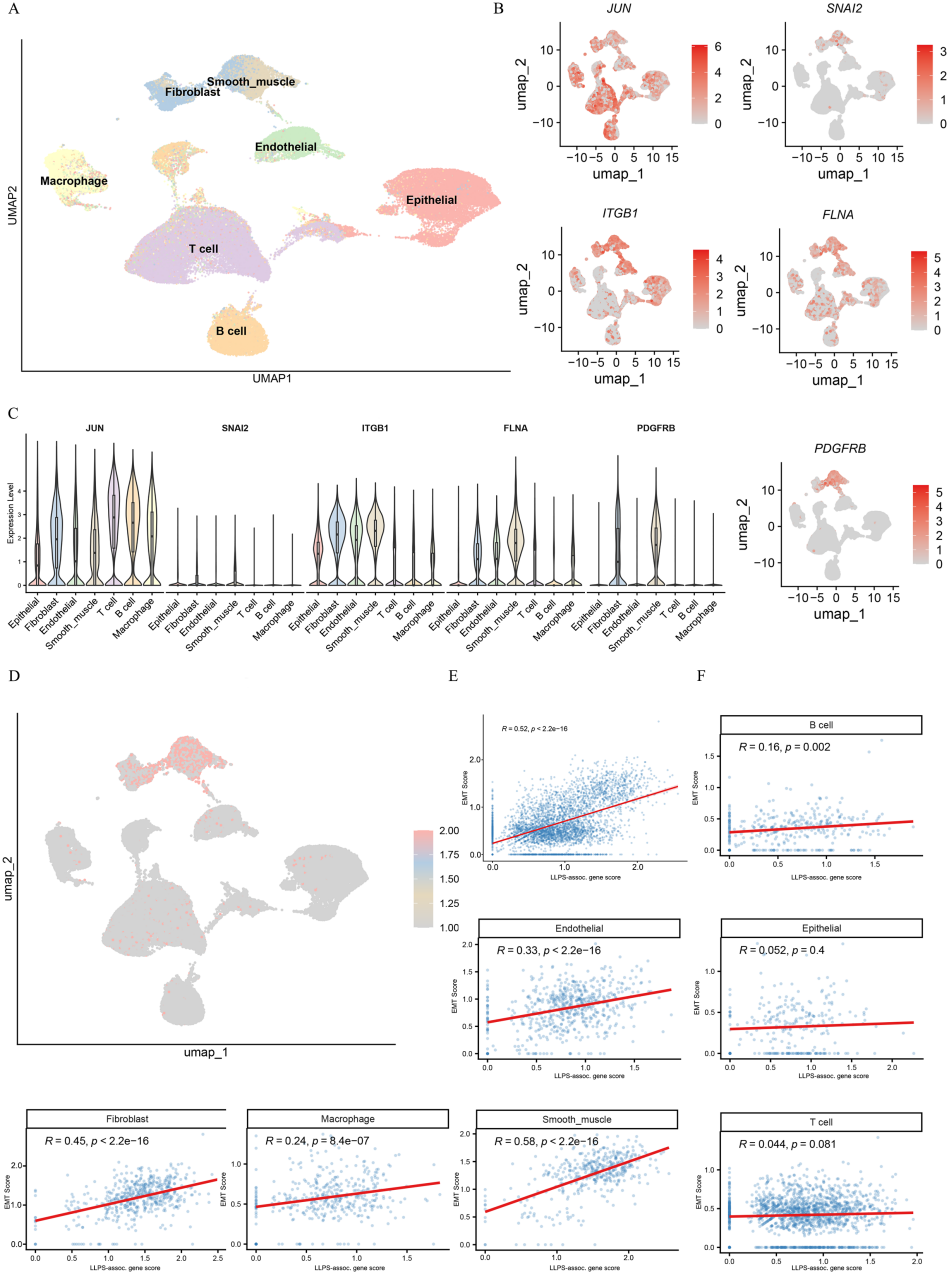
Single-cell landscape of LLPS-EMT axis in BLCA. **(A)** UMAP visualization of 74, 416 cells from 9 GSE222315 samples, colored by cell type. Seven major populations were identified: epithelial cells (pink), T cells (purple), B cells (orange), macrophages (yellow), fibroblasts (blue), endothelial cells (green), and smooth muscle cells (beige). **(B)** Feature plots showing the expression distribution of five core LLPS genes (JUN, SNAI2, ITGB1, FLNA, PDGFRB) across cell types. Color intensity represents expression level (red = high, gray = low). **(C)** Violin plots quantifying cell type-specific expression levels of the five core genes. **(D)** Distribution of the LLPS-associated gene expression score (LLPS-assoc. gene score) calculated as the mean expression of the five core genes. UMAP projection colored by LLPS-assoc. gene score, with red indicating high expression. Smooth muscle cells and fibroblasts show dense red regions, while other cell types exhibit scattered red points. **(E)** Scatter plot showing correlation between the LLPS-assoc. gene score and EMT score across all cell types (r = 0.52, p < 2.2e-16). Blue points represent individual cells; red line shows linear regression with 95% confidence interval. **(F)** Faceted scatter plots showing correlation between the LLPS-assoc. gene score and EMT score in each of the seven major cell types. For hematopoietic-derived immune cells (T cells, B cells, macrophages), the EMT score reflects the expression of EMT-associated genes rather than a bona fide EMT program. Pearson correlation coefficients (r) and p-values are indicated.

To investigate the cellular distribution of the core genes, we analyzed scRNA-seq data from 74, 416 cells across nine BLCA samples. UMAP visualization identified seven major cell populations, including epithelial cells, T cells, B cells, macrophages, fibroblasts, endothelial cells, and smooth muscle cells (**Figure 4A**). Feature plots and violin plots revealed that while JUN, SNAI2, ITGB1, and FLNA were broadly expressed across multiple lineages, PDGFRB exhibited a more restricted expression pattern, primarily localized in smooth muscle cells (**Figures 4B, C**). We then calculated an LLPS-associated gene expression score (LLPS-assoc. gene score) based on the mean expression of these five core genes. High expression of these genes was notably concentrated in smooth muscle cells and fibroblasts, though scattered cells with high expression of these genes were also observed within other populations (**Figure 4D**). Globally, the LLPS-assoc. gene score showed a significant positive correlation with the EMT score across the entire single-cell dataset (**Figure 4E**). Further faceted analysis showed that positive correlations between the LLPS-assoc. gene score and the EMT score were observed across all seven cell types (**Figure 4F**). For hematopoietic-derived immune cells (T cells, B cells, macrophages), the EMT score reflects the expression of EMT-associated genes rather than an active epithelial-mesenchymal transition program (**Figure 4F**).

### 3.5 Dynamic regulation of LLPS and ferroptosis in epithelial heterogeneity

**Figure 5:**
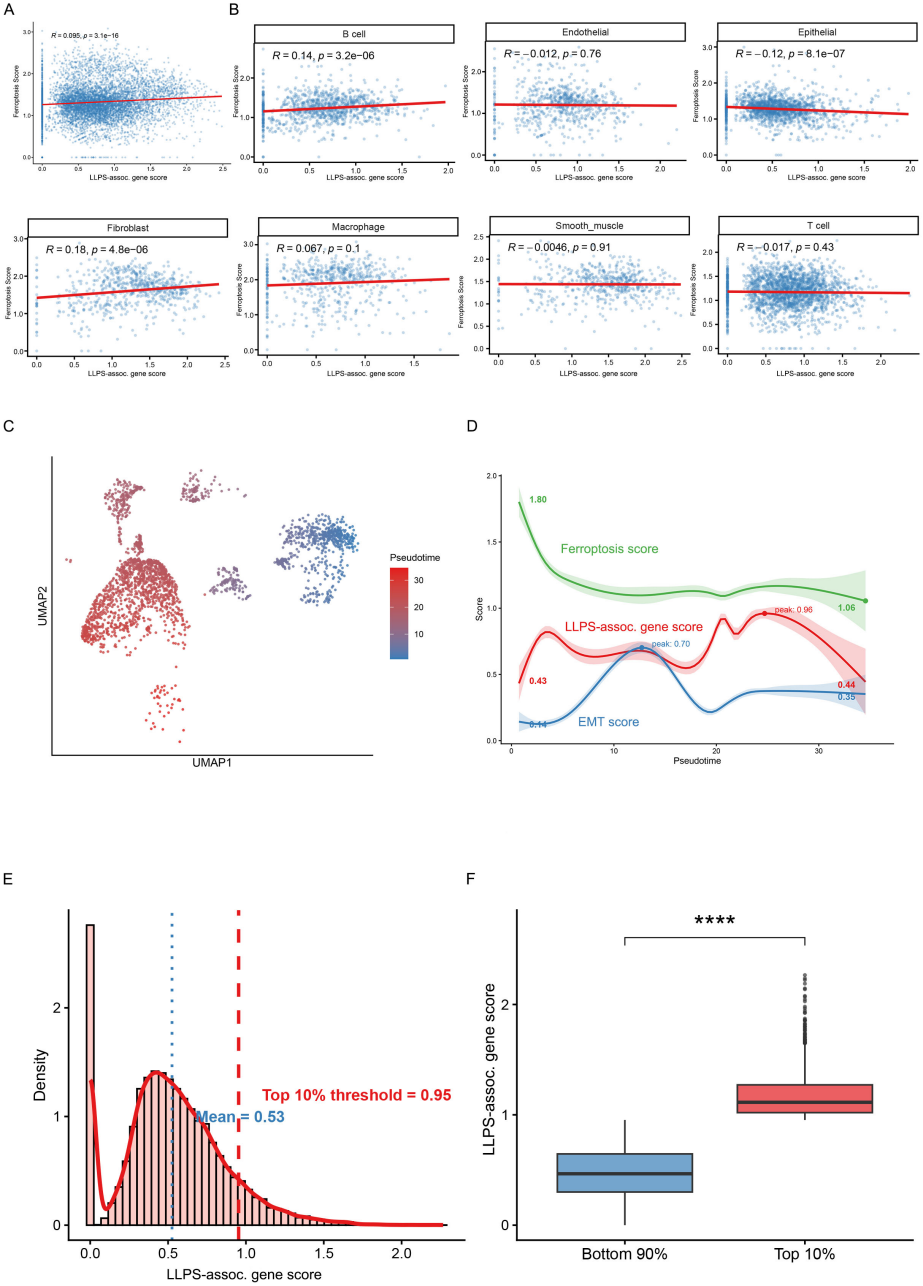
LLPS-ferroptosis relationship and epithelial heterogeneity in BLCA. **(A)**Scatter plot showing correlation between the LLPS-assoc. gene score and ferroptosis score across all cell types. Blue points represent individual cells; red line shows linear regression with 95% confidence interval. **(B)** Faceted scatter plots showing correlation between the LLPS-assoc. gene score and ferroptosis score in each of the seven major cell types. Epithelial cells exhibit a negative correlation. **(C)** Pseudotime trajectory analysis of epithelial and smooth muscle cells. UMAP plot colored by pseudotime, showing progression from low (blue) to high (red) pseudotime. **(D)** Smooth curves showing dynamic changes in the LLPS-assoc. gene score, EMT score, and ferroptosis score along pseudotime. Upregulation of LLPS-associated genes coincides with rapid ferroptosis suppression, followed by EMT induction. **(E)** Distribution of the LLPS-assoc. gene scores within epithelial cells. Histogram and density plot showing the distribution among 17, 861 epithelial cells. Vertical lines indicate the mean (blue dotted) and top 10% threshold (red dashed). **(F)** Comparison of the LLPS-assoc. gene scores between top 10% and bottom 90% epithelial cells. Box plot showing that the top 10% epithelial cells (n = 1, 786) exhibits significantly higher LLPS-assoc. gene scores compared to the bottom 90% (n = 16, 075). Wilcoxon test, ****p < 0.0001.

A global positive correlation was observed between the LLPS-assoc. gene score and the ferroptosis score across the single-cell dataset (**Figure 5A**). However, faceted analysis revealed a distinct negative correlation specifically within the epithelial cell population (**Figure 5B**). Pseudotime trajectory analysis of epithelial and smooth muscle cells demonstrated a clear evolutionary progression from a low-pseudotime state to a specialized high-pseudotime state (**Figure 5C**). Interestingly, dynamic expression profiling along the trajectory showed that upregulation of LLPS-associated genes coincides with rapid ferroptosis suppression, which subsequently precedes the full induction of the EMT program (**Figure 5D**). This observed temporal pattern suggests that the transcriptional program of LLPS-associated genes is closely linked to ferroptosis resistance during the cellular transition toward a mesenchymal-like state. Heterogeneity analysis within the epithelial compartment further revealed a subset of cells with high expression of LLPS-associated genes (**Figure 5E**). Specifically, the top 10% of epithelial cells (n = 1, 786) displayed significantly higher LLPS-assoc. gene scores compared to the bottom 90% (n = 16, 075), with expression levels approaching those typically observed in smooth muscle cells (**Figure 5F**). This subset with elevated expression of LLPS-associated genes may represent cells undergoing EMT, potentially contributing to the robust prognostic power of our 5-gene signature. Collectively, these findings establish this specialized epithelial subset as a key focus for investigating therapy resistance and the complex spatial organization of bladder cancer.

### 3.6 Spatial transcriptomics and histological validation of PDGFRB condensates in bladder cancer

**Figure 6:**
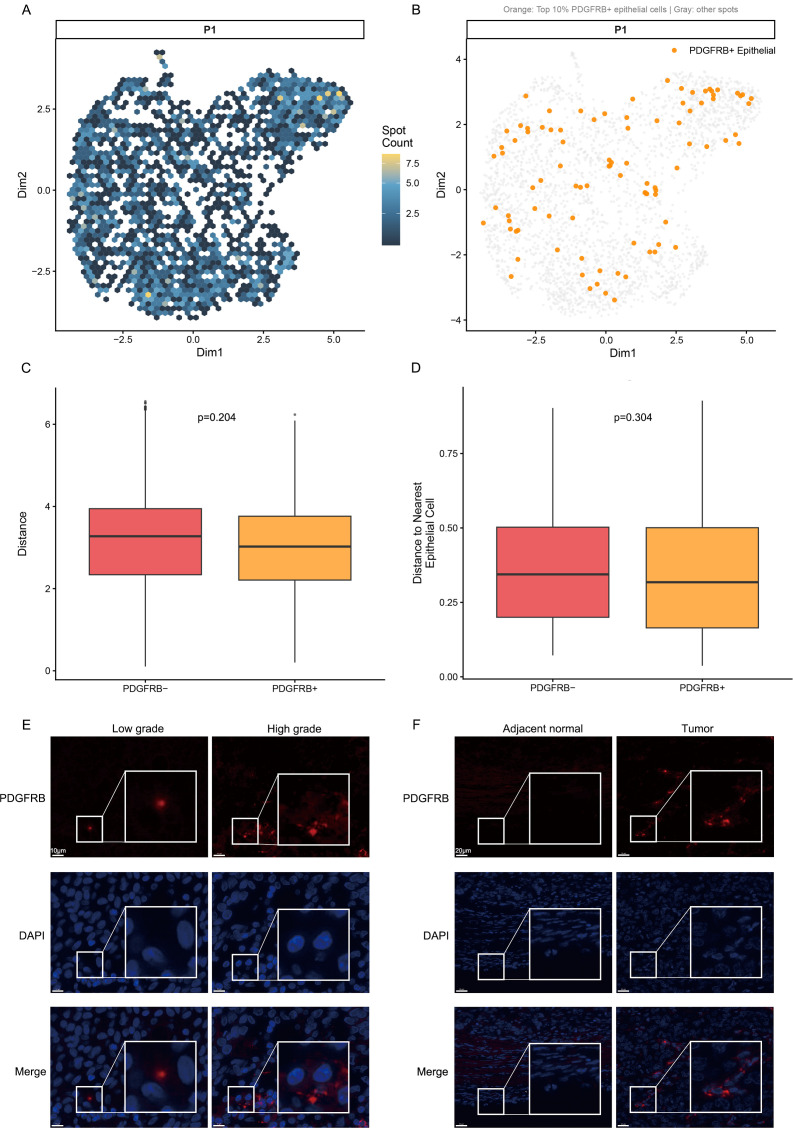
Spatial transcriptomics and histological validation of PDGFRB condensates in bladder cancer. **(A)** Spatial transcriptome layout. Hexbin plots showing spot density distribution across four BLCA tissue sections (GSE247629). Color intensity represents log-transformed spot count per hexagon. **(B)** Spatial distribution of PDGFRB+ epithelial cells. Gray dots represent all other spots; orange dots indicate PDGFRB+ epithelial cells, defined as the top 10% of PDGFRB-expressing epithelial cells (n = 59 spots across all samples). **(C)** Distance to epithelial center. Boxplot comparing the distance of PDGFRB+ (orange, n = 59) and PDGFRB- (red, n = 1, 878) epithelial cells to the epithelial center. Wilcoxon rank-sum test, p = 0.204. **(D)** Nearest neighbor distance. Boxplot comparing the distance to the nearest neighboring epithelial cell for PDGFRB+ (n = 59) and PDGFRB- (n = 59, randomly sampled) epithelial cells. Wilcoxon rank-sum test, p = 0.304. **(E)** Immunofluorescence staining of PDGFRB (red) in human bladder cancer tissues from low-grade (left) and high-grade (right) tumors. Nuclei were counterstained with DAPI (blue). Insets show magnified views. Scale bar, 10 μm. **(F)** Immunofluorescence staining of PDGFRB (red) in mouse orthotopic bladder tumors comparing normal urothelium (left) and tumor tissue (right). Nuclei were counterstained with DAPI (blue). Scale bar, 20 μm.

To validate the spatial localization of the PDGFRB+ epithelial cells, we analyzed spatial transcriptomics data from four BLCA tissue sections (GSE247629). The spatial architecture and spot layout were first established to provide a topographical context (**Figure 6A**). We specifically visualized the PDGFRB+ epithelial cells, defined as the top 10% of PDGFRB-expressing epithelial spots (n = 59), across the tissue sections (**Figure 6B**). Quantitative spatial analysis revealed that these PDGFRB+ cells did not exhibit preferential sequestration toward either the tumor core or the invasive margin, as evidenced by comparable distances to the epithelial center between PDGFRB+ and PDGFRB- populations (**Figure 6C**). Furthermore, nearest neighbor distance analysis confirmed that these cells were not spatially aggregated into clusters but were instead dispersedly distributed throughout the epithelial compartment (**Figure 6D**). These findings suggest that PDGFRB+ cells may function as decentralized molecular hubs within the tumor architecture, contributing to the broader spatial heterogeneity of bladder cancer.

To further examine PDGFRB at the protein level, we performed immunofluorescence staining on clinical bladder cancer specimens. In low-grade tumors, PDGFRB exhibited occasional small puncta. In marked contrast, high-grade tumors displayed prominent, discrete punctate structures throughout the cytoplasm, reminiscent of biomolecular condensates (**Figure 6E**). Consistently, in a mouse orthotopic bladder cancer model, PDGFRB puncta were readily detected in tumor tissues, whereas adjacent normal urothelium showed sparse PDGFRB signal with no discernible puncta (**Figure 6F**). These histological observations provide direct evidence that endogenous PDGFRB forms condensate-like structures in bladder cancer tissues, supporting its predicted phase separation capacity.

### 3.7 PDGFRB phase separation capacity and its role in suppressing ferroptosis in BLCA cells

**Figure 7:**
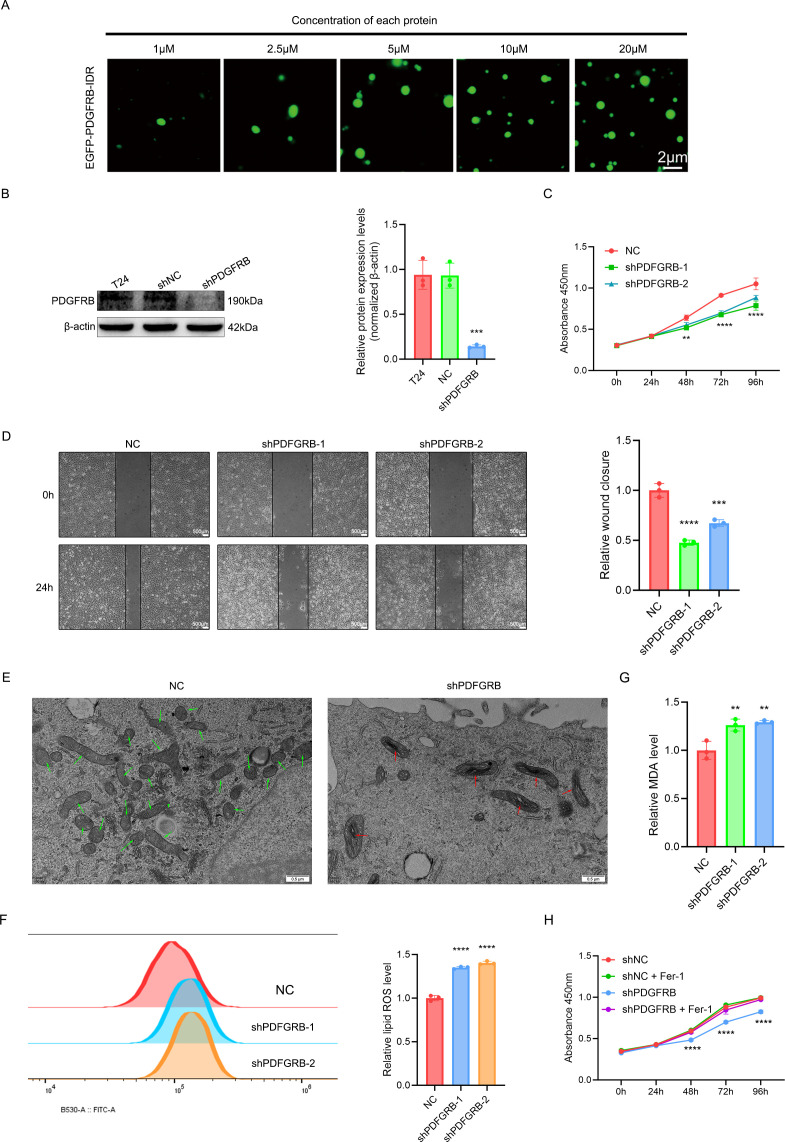
PDGFRB promotes proliferation, migration, and suppresses ferroptosis in bladder cancer cells. **(A)** *In vitro* droplet formation assay of purified recombinant EGFP-PDGFRB-IDR (residues 951–1089) at the indicated concentrations, imaged by confocal microscopy. Scale bar, 2 μm. **(B)** Western Blot analysis of PDGFRB protein levels in parental T24, shNC (scramble control), and shPDGFRB cells using anti-PDGFRB antibody, with β-actin as loading control. **(C)** Cell proliferation. CCK-8 assay showing cell viability of shNC and shPDGFRB T24 cells at indicated time points (0, 24, 48, 72, 96 h). **(D)** Cell migration. Left: Representative images of wound healing assay at 0 and 24 h. Scale bar, 500 μm. Right: Quantification of wound closure rate. **(E)** Mitochondrial morphology. Transmission electron microscopy images showing mitochondrial morphology in shNC and shPDGFRB T24 cells. Green arrows indicate normal mitochondria with intact cristae; red arrows indicate ferroptotic mitochondria with shrinkage and membrane densification. Scale bar, 0.5 μm. **(F)** Lipid peroxidation. Left: Ridge plot showing C11-BODIPY fluorescence intensity distribution in shNC and shPDGFRB T24 cells measured by flow cytometry. Right: Quantification of mean fluorescence intensity. **(G)** MDA levels. Malondialdehyde (MDA) levels in shNC and shPDGFRB T24 cells measured by MDA assay kit. **(H)** Ferroptosis rescue assay. CCK-8 assay showing cell viability of shNC and shPDGFRB T24 cells with or without Ferrostatin-1 (Fer-1, 2 μM) at indicated time points (0, 24, 48, 72, 96 h). All experiments were performed independently at least three times. For **(C, D, F, G)**, each independent experiment included two technical replicates (labeled shPDGFRB-1 and shPDGFRB-2) of the same shPDGFRB construct. Data are presented as mean ± s.d. For **(B)**, significance was calculated by one-way ANOVA. For **(C, D, F, G)**, significance was calculated by unpaired two-tailed Student's t test. For **(H)**, significance was calculated by two-way ANOVA. **p < 0.01, ***p < 0.001, ****p < 0.0001.

To determine whether the intrinsically disordered region (IDR) of PDGFRB possesses intrinsic phase separation ability, we purified recombinant EGFP-PDGFRB-IDR (residues 951-1089) and performed an *in vitro* droplet formation assay. The purified protein formed spherical droplets in a concentration-dependent manner, with increased droplet formation observed as the protein concentration increased from 1 to 20 μM (**Figure 7A**). This result establishes that the PDGFRB IDR can independently drive liquid-liquid phase separation under physiologically relevant crowding conditions.

We next investigated the functional consequences of PDGFRB in bladder cancer cells. Stable PDGFRB knockdown T24 cell lines were generated. Western Blot analysis confirmed endogenous PDGFRB expression in parental T24 cells and shNC control cells, and its effective depletion in shPDGFRB cells (**Figure 7B**). Functional assays revealed that PDGFRB depletion significantly impaired cell viability starting from 48 hours (**Figure 7C**) and markedly inhibited migratory capacity in wound healing assays (**Figure 7D**). Crucially, transmission electron microscopy revealed features characteristic of ferroptosis, including shrunken mitochondria with increased membrane density and loss of cristae (**Figure 7E**). These morphological findings were corroborated by biochemical evidence, as shPDGFRB cells exhibited significantly elevated lipid peroxidation measured by C11-BODIPY (**Figure 7F**) and increased malondialdehyde (MDA) levels (**Figure 7G**). To definitively establish ferroptosis as the mode of cell death, we performed a functional rescue assay using the specific lipid ROS scavenger Ferrostatin-1. Treatment with Fer-1 (2 μM) significantly restored cell viability in shPDGFRB cells (**Figure 7H**), confirming that PDGFRB depletion triggers ferroptosis-dependent cell death. Collectively, these results demonstrate that PDGFRB possesses intrinsic phase separation capacity mediated by its IDR and functions as a critical suppressor of ferroptosis in bladder cancer cells.

### 3.8 Immune infiltration landscape associated with the 5-gene signature

**Figure 8:**
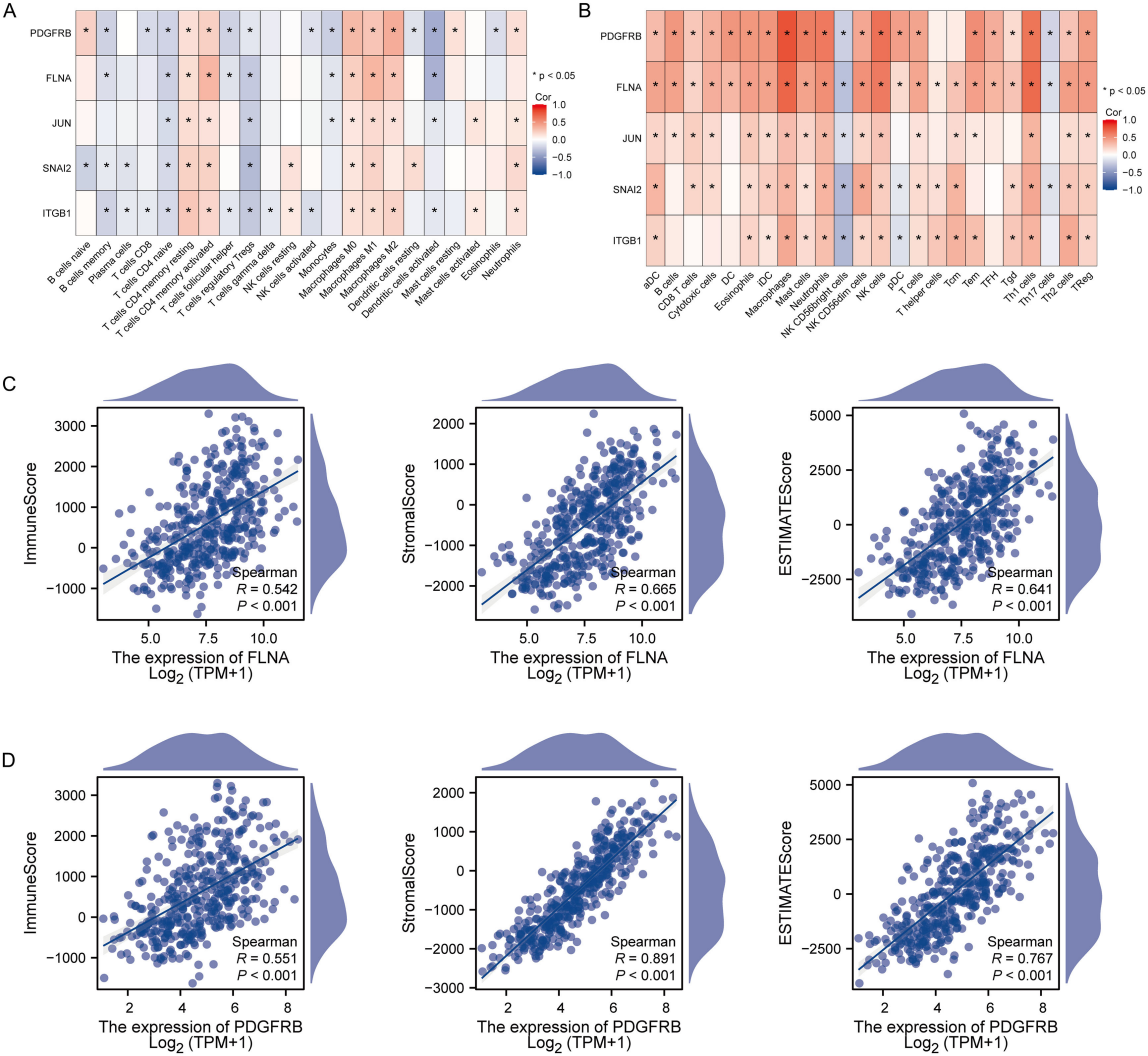
Immune infiltration landscape associated with the 5-gene signature. **(A)** Correlation heatmap showing associations between the five prognostic genes and the relative proportions of 22 tumor-infiltrating immune cell types, as calculated by CIBERSORT using the LM22 signature matrix. Red indicates positive correlation, blue indicates negative correlation. *p < 0.05. **(B)** Correlation heatmap showing associations between the five prognostic genes and the enrichment levels of 24 immune cell groups, as assessed by ssGSEA using immune-related gene sets from MSigDB. Red indicates positive correlation, blue indicates negative correlation. *p < 0.05. **(C)** Scatter plots showing correlations between FLNA and immune score, stromal score, and ESTIMATE score calculated by the ESTIMATE method. Spearman correlation coefficients **(r)** and p-values are indicated. **(D)** Scatter plots showing correlations between PDGFRB and immune score, stromal score, and ESTIMATE score calculated by the ESTIMATE method. Spearman correlation coefficients (r) and p-values are indicated.

Both CIBERSORT and ssGSEA analyses demonstrated that the five prognostic genes were linked to the infiltration patterns of tumor-associated immune cells (**Figures 8A, B**). Moreover, FLNA and PDGFRB showed significant correlations with immune score, stromal score, and ESTIMATE score, suggesting their potential involvement in shaping the tumor immune microenvironment (**Figure 8C, D**).

## 4 Discussion

The convergence of LLPS-associated gene networks, EMT, and ferroptosis represents a nascent paradigm in cancer biology. Our study identifies a 5-gene transcriptomic signature (PDGFRB, FLNA, JUN, SNAI2, ITGB1) that is associated with known phase-separating networks and effectively stratifies BLCA prognosis. The LLPS-associated gene expression score derived from this signature reflects the transcriptional activity of genes whose protein products are predicted or annotated to participate in LLPS, as supported by database annotation and IDR content analysis (8, 41, 52). We emphasize that this score provides a transcriptomic readout and should be clearly distinguished from direct biophysical measurements of protein condensation. The high IDR content of FLNA (52%) and PDGFRB (45%), together with their DrLLPS classification as a regulator and client, respectively, suggests their capacity for participation in phase-separating networks. Beyond these in silico predictions, our immunofluorescence data provide direct histological evidence that endogenous PDGFRB forms condensate-like puncta in both human and mouse bladder cancer tissues (**Figures 6E, F**), and *in vitro* reconstitution experiments confirm that the IDR of PDGFRB can independently drive phase separation (**Figure 7A**). These experimental results substantiate the phase separation competence originally inferred from our bioinformatic analyses. Furthermore, the 5-gene signature was externally validated in an independent BLCA cohort (GSE13507, n = 165), confirming its prognostic value beyond the TCGA training dataset. Our functional validation further demonstrates that PDGFRB knockdown impairs cell proliferation and migration, and triggers ferroptosis as confirmed by characteristic mitochondrial damage, elevated lipid peroxidation, and rescue by Ferrostatin-1 (**Figures 7E–H**). These findings position PDGFRB as the most clinically actionable core node within this regulatory network.

The identification of PDGFRB as a ferroptosis suppressor extends its known roles in therapy resistance and invasion in bladder cancer (21, 22). While prior studies linked PDGFRB to PI3K/Akt signaling in gastric cancer ferroptosis (53), our findings independently demonstrate that PDGFRB knockdown directly induces hallmark mitochondrial damage in BLCA cells. The molecular mechanisms by which PDGFRB suppresses ferroptosis in bladder cancer remain to be fully elucidated, but several plausible pathways can be proposed. Activation of the PI3K/Akt signaling axis downstream of PDGFRB may upregulate antioxidant defense programs, such as GPX4 expression or SREBP1-mediated lipid metabolism. These programs could limit lipid peroxidation and thereby suppress ferroptotic cell death. Alternatively, PDGFRB signaling may modulate iron homeostasis or enhance the glutathione biosynthesis pathway to maintain redox balance. Future studies using pharmacological inhibitors of specific downstream effectors, combined with metabolomic and transcriptomic profiling, will be required to define the precise biochemical links between PDGFRB phase separation and ferroptosis suppression. Single-cell and spatial analyses further revealed that PDGFRB+ epithelial cells exhibit a unique dispersed spatial pattern rather than clustering at the tumor invasive margin. This non-localized distribution, coupled with elevated expression of LLPS-associated genes in these cells, suggests that they may function as decentralized signaling hubs that influence the microenvironment through paracrine mechanisms. Both CIBERSORT and ssGSEA analyses further demonstrated that the five prognostic genes were linked to the infiltration patterns of tumor-associated immune cells, and FLNA and PDGFRB showed significant correlations with immune, stromal, and ESTIMATE scores. These observations raise the possibility that elevated expression of LLPS-associated genes in these cells may influence the immune contexture of the tumor microenvironment, potentially through paracrine mechanisms or by modulating the expression of immune-regulatory factors. Notably, phase separation has recently emerged as a new biophysical principle in organizing immune signaling components into large signaling clusters with fluidic properties, providing mechanistic insights into how biomolecular condensates may shape immune responses (54). Within this framework, PDGFRB-associated condensates observed in bladder cancer tissues could potentially remodel the tumor immune microenvironment through several non-mutually exclusive mechanisms, including the regulation of immunomodulatory cytokine secretion by tumor cells, spatial reorganization of immune signaling molecules at the tumor–immune interface, and modulation of antigen presentation pathways. However, the functional and therapeutic relevance of these correlations remains to be experimentally validated, and dedicated studies are required to determine whether PDGFRB condensates directly contribute to immune evasion in bladder cancer.

Our finding that PDGFRB, a transmembrane receptor, exhibits LLPS capacity is consistent with the emerging paradigm that membrane proteins can undergo phase separation at two-dimensional membrane surfaces, a phenomenon termed ‘prewetting’ that was recently demonstrated for the ER-resident protein PDZD8 (18). We note, however, an important distinction: while the *in vitro* droplet formation of purified PDGFRB-IDR demonstrates the intrinsic phase separation potential of this isolated domain, it does not directly confirm that the full-length, membrane-anchored receptor undergoes bona fide LLPS at the plasma membrane under physiological conditions. The IDR sequence alone suggests LLPS capacity but requires experimental verification in the context of the native, full-length protein. Therefore, future studies employing live-cell imaging of full-length PDGFRB, such as fluorescence recovery after photobleaching (FRAP) or optogenetic approaches, will be required to confirm physiological LLPS in the native membrane environment. The functional connection between LLPS and ferroptosis observed in our study aligns with recent reports. For instance, phase separation of FSP1 promotes ferroptosis (35), while phosphorylation-driven PRDX1 phase separation suppresses its peroxidase activity, leading to ferroptosis in endothelial cells (55). Our findings extend this paradigm to PDGFRB, identifying it as a novel LLPS-associated ferroptosis suppressor in bladder cancer. Our pseudotime trajectory analysis further revealed that the transcriptional upregulation of LLPS-associated genes coincides with ferroptosis suppression during the transition toward mesenchymal-like states. This temporal sequence raises the hypothesis that LLPS-associated gene programs may contribute to a cellular state that facilitates ferroptosis resistance during EMT, potentially protecting transitioning cells from oxidative cell death as they acquire metastatic competence. We note that the EMT scores calculated for hematopoietic-derived immune cells reflect the expression of EMT-associated gene sets rather than a canonical EMT program, and interpretations involving these populations should be made with this caveat in mind.

Despite these insights, several limitations should be acknowledged. First, our functional experiments were primarily conducted in a single cell line (T24). Although key findings were corroborated in clinical specimens and *in vivo* models, validation in additional BLCA cell lines would further strengthen the generalizability of our conclusions. Second, our prognostic signature was externally validated in only one independent cohort (GSE13507). Validation in additional diverse patient populations is warranted before clinical application. Third, the precise biochemical mechanisms by which PDGFRB phase-separates, and whether these condensates directly mediate ferroptosis suppression, require further investigation using live-cell imaging of condensate dynamics, fluorescence recovery after photobleaching (FRAP), and 1, 6-hexanediol sensitivity assays. Fourth, our functional validation relied on loss-of-function approaches (shRNA-mediated knockdown). Complementary gain-of-function studies, such as overexpression of wild-type PDGFRB or its IDR-deleted mutant, would provide more direct causal evidence that PDGFRB actively drives ferroptosis suppression. These experiments represent an important direction for future investigation.

We also acknowledge that for four of the five signature genes (ITGB1, FLNA, c-JUN, and SNAI2), our current evidence remains at the level of transcriptomic correlation with LLPS-associated networks. The phase separation of these proteins is known to depend on specific cellular contexts that are not captured by mRNA abundance measurements, including ECM-mediated mechanical tension for ITGB1, intact actin cytoskeleton for FLNA, post-translational modifications for c-JUN, and active condensate formation beyond transcriptional upregulation for SNAI2. For these four proteins, dedicated biophysical assays are required to determine whether they form functional condensates in BLCA cells. Our integrated multi-omics analysis therefore identifies these genes as components of a transcriptionally coordinated LLPS-associated network, rather than empirically validated phase-separating proteins. The therapeutic efficacy of combining PDGFRB inhibitors with ferroptosis inducers also warrants further validation in matched patient cohorts and *in vivo* models.

In conclusion, this study identifies a five-gene transcriptomic signature that stratifies BLCA prognosis and is associated with LLPS-, EMT-, and ferroptosis-related gene networks. Functional experiments validate PDGFRB as a suppressor of ferroptosis, and new histological and *in vitro* evidence demonstrates the phase-separation capacity of its IDR domain. While the prognostic signature is derived from transcriptomic correlations, these findings provide a hypothesis-generating framework for future mechanistic investigations into LLPS-driven ferroptosis resistance in bladder cancer. Targeting the PDGFRB axis may thus offer a promising strategy for ferroptosis-based intervention in aggressive bladder cancer.

## Data Availability

The data that support the findings of this study are openly available in public repositories. Transcriptomic and clinical data for bladder cancer patients are available from The Cancer Genome Atlas (TCGA) database at https://portal.gdc.cancer.gov. The external validation cohort (GSE13507) and single-cell RNA sequencing data (GSE222315) and spatial transcriptomics data (GSE247629) were downloaded from the Gene Expression Omnibus (GEO) database at https://www.ncbi.nlm.nih.gov/geo/. The LLPS-related gene set is available from the DrLLPS database at http://llps.biocuckoo.cn/, the ferroptosis-related gene set from FerrDb V3 at http://www.zhounan.org/ferrdb/, and the EMT-related gene set from the Molecular Signatures Database (MSigDB) at https://www.gsea-msigdb.org/gsea/msigdb/.
